# An artificial intelligence-based bone age assessment model for Han and Tibetan children

**DOI:** 10.3389/fphys.2024.1329145

**Published:** 2024-02-15

**Authors:** Qixing Liu, Huogen Wang, Cidan Wangjiu, Tudan Awang, Meijie Yang, Puqiong Qiongda, Xiao Yang, Hui Pan, Fengdan Wang

**Affiliations:** ^1^ Department of Radiology, Peking Union Medical College Hospital, Chinese Academy of Medical Sciences and Peking Union Medical College, Beijing, China; ^2^ College of Computer Science and Technology, Zhejiang University, Hangzhou, China; ^3^ Department of Radiology, Tibet Autonomous Region People’s Hospital, Lhasa, China; ^4^ Department of Radiology, People’s Hospital of Nyima County, Nagqu, China; ^5^ Department of Radiology, People’s Hospital of Nagqu, Nagqu, China; ^6^ Department of Ultrasound, Peking Union Medical College Hospital, Chinese Academy of Medical Sciences and Peking Union Medical College, Beijing, China; ^7^ Department of Endocrinology, Peking Union Medical College Hospital, Chinese Academy of Medical Sciences and Peking Union Medical College, Beijing, China

**Keywords:** bone age, artificial intelligence, deep learning, Tibetan, Han, children

## Abstract

**Background:** Manual bone age assessment (BAA) is associated with longer interpretation time and higher cost and variability, thus posing challenges in areas with restricted medical facilities, such as the high-altitude Tibetan Plateau. The application of artificial intelligence (AI) for automating BAA could facilitate resolving this issue. This study aimed to develop an AI-based BAA model for Han and Tibetan children.

**Methods:** A model named “EVG-BANet” was trained using three datasets, including the Radiology Society of North America (RSNA) dataset (training set *n* = 12611, validation set *n* = 1425, and test set *n* = 200), the Radiological Hand Pose Estimation (RHPE) dataset (training set *n* = 5491, validation set *n* = 713, and test set *n* = 79), and a self-established local dataset [training set *n* = 825 and test set *n* = 351 (Han *n* = 216 and Tibetan *n* = 135)]. An open-access state-of-the-art model BoNet was used for comparison. The accuracy and generalizability of the two models were evaluated using the abovementioned three test sets and an external test set (*n* = 256, all were Tibetan). Mean absolute difference (MAD) and accuracy within 1 year were used as indicators. Bias was evaluated by comparing the MAD between the demographic groups.

**Results:** EVG-BANet outperformed BoNet in the MAD on the RHPE test set (0.52 vs. 0.63 years, *p* < 0.001), the local test set (0.47 vs. 0.62 years, *p* < 0.001), and the external test set (0.53 vs. 0.66 years, *p* < 0.001) and exhibited a comparable MAD on the RSNA test set (0.34 vs. 0.35 years, *p* = 0.934). EVG-BANet achieved accuracy within 1 year of 97.7% on the local test set (BoNet 90%, *p* < 0.001) and 89.5% on the external test set (BoNet 85.5%, *p* = 0.066). EVG-BANet showed no bias in the local test set but exhibited a bias related to chronological age in the external test set.

**Conclusion:** EVG-BANet can accurately predict the bone age (BA) for both Han children and Tibetan children living in the Tibetan Plateau with limited healthcare facilities.

## 1 Introduction

Bone age (BA), an indicator of skeletal development, objectively reflects the growth and bone maturity of children as compared to chronological age (CA) (Creo and Schwenk, 2017). BA is mainly determined from the radiographs of the left hand and wrist using the Greulich–Pyle (GP) (Greulich and Idell Pyle, 1959) or Tanner–Whitehouse method (Tanner, 1962; Tanner, 1983; Tanner et al., 2001). The GP method is generally favored for its simplicity and practicality and is, therefore, widely applied in clinical settings. The manual evaluation of BA, however, heavily relies on the reviewer’s experience, resulting in notable intra- and inter-observer variations. The considerable time invested in training clinical reviewers poses difficulties in implementing BA assessment (BAA) in areas with limited medical resources (Wang et al., 2020). In recent years, artificial intelligence (AI) and deep learning have emerged as new possibilities of automating BAA (Prokop-Piotrkowska et al., 2021), leading to the development of various autonomous approaches (Kim et al., 2017; Lee et al., 2017; Spampinato et al., 2017; Larson et al., 2018; Mutasa et al., 2018; Escobar et al., 2019; Ren et al., 2019; Zhou et al., 2020; Nguyen et al., 2022; Yang et al., 2023) that effectively address the drawbacks of traditional manual methods, thereby achieving a reduction in interpretation time and variability while concurrently enhancing accuracy (Tajmir et al., 2019; Eng et al., 2021; Lee et al., 2021).

However, concerns have been raised regarding the generalizability and bias of AI BAA systems (Beheshtian et al., 2023; Larson, 2023). The AI BAA models were primarily developed using certain population groups, such as the North American (Lee et al., 2017; Larson et al., 2018; Mutasa et al., 2018), Korean (Kim et al., 2017), and Chinese population of Han ethnicity (Ren et al., 2019; Zhou et al., 2020; Yang et al., 2023). China is a culturally diverse nation encompassing various ethnic groups; these include the Tibetan people who mainly reside in the Tibetan Plateau, which is located at 4000 m above sea level (asl) and has limited medical resources (Harris et al., 2001; Wang et al., 2021). To the best of our knowledge, there is currently no AI BAA model developed for Tibetan children. The constraints of training the population make the existing models potentially unsuitable for application beyond specific populations on which they were trained. Moreover, because of the limitation of models that may focus more on populations of specific groups, there is a possibility of introducing bias based on variables such as age, gender, and ethnicity. Systematic bias, unlike random errors, can be resolved through compensatory techniques such as sampling adjustments, augmentation, calibration, weighting adjustments, and the inclusion of additional input variables into the algorithm (Larson, 2023).

To address these concerns, we developed a fully automated AI BAA system termed “EVG-BANet” using data from three distinct datasets, one of which was our self-established dataset that includes both Han and Tibetan populations. Our model incorporates both gender and ethnicity information as independent variables into global and local visual features extracted by deep learning. To the best of our knowledge, EVG-BANet is the first model to achieve this. Based on evaluation using four different datasets, EVG-BANet outperformed BoNet, the current state-of-the-art model, in terms of accuracy and generalizability.

## 2 Methods

### 2.1 Dataset description

This multicenter study was approved by the Institutional Review Board of Peking Union Medical College Hospital (I-22PJ458), and the informed consent requirement was waived because of the retrospective nature of the study.

The EVG-BANet model was trained using data from three different datasets of radiographs acquired from the left hand and wrist of children: the Radiology Society of North America (RSNA) dataset (Halabi et al., 2019), the Radiological Hand Pose Estimation (RHPE) dataset (Escobar et al., 2019), and our self-established local dataset (Table 1). The RSNA dataset included 14236 images from Lucile Packard Children’s Hospital at Stanford University and Children’s Hospital Colorado; of these images, 12611 radiographs were randomly selected as the training set and 1425 radiographs were used as the validation set, with an additional test set (*n* = 200). The RHPE dataset comprised 6283 images that were divided into three sets: a training set (*n* = 5491), validation set (*n* = 713), and test set (*n* = 79). The local dataset included BA radiographs from two medical centers at different altitudes: Peking Union Medical College Hospital in Beijing at 43.5 m asl (*n* = 745, all were Han) (Wang et al., 2020) and Tibet Autonomous Region People’s Hospital in Lhasa at 3650 m asl (*n* = 431, including 114 Han children and 317 Tibetan children) (Wang et al., 2021). In total, 1176 cases were included in the local dataset and were randomly divided into the training set (*n* = 825) and test set (*n* = 351).

**TABLE 1:** Summary information for the training, validation, and test datasets.

Variable	Number of males	Number of females	Total	Chronologic age (years)	Bone age (years)
Training set					
RSNA	6833	5778	12611	/	10.61 ± 3.43
RHPE	2372	3119	5491	10.35 ± 3.24	10.25 ± 3.46
Local	473	352	825	11.79 ± 3.91	11.20 ± 3.70
Validation set					
RSNA	773	652	1425	/	10.60 ± 3.48
RHPE	306	407	713	10.30 ± 3.24	10.24 ± 3.46
Test set					
RSNA	100	100	200	/	11.01 ± 3.36
RHPE	38	41	79	10.26 ± 3.28	10.34 ± 3.20
Local	201	150	351	11.71 ± 3.98	11.30 ± 3.97
External	153	103	256	11.30 ± 5.30	9.79 ± 5.29

The RSNA dataset does not include information about the chronological age.

Additionally, an external dataset comprising BA radiographs from Tibetan children at Nyima County People’s Hospital in Nagqu at 4500 m asl was included as a test set (*n* = 256, all were Tibetan). The detailed inclusion and exclusion criteria are provided in Supplementary Table S1.

### 2.2 Reference BA standard

The reference BA standards provided with the RSNA and RHPE datasets were used as the ground truth for training. The ground truth BA standards for the local dataset and the external test set were determined by two experienced doctors (a radiologist with 10 years of experience and an endocrinologist with 15 years of experience in BA reading) (Beheshtian et al., 2023) through mutual consensus using the GP method (Greulich and Idell Pyle, 1959). The readers were aware of only the age and gender of the patient and were blinded to ethnicity and clinical details. For any disagreement, a third reviewer, an endocrinologist specialized in child growth and development with over 20 years of experience in BA reading, was consulted. The atlas used for the assessment was “Skeletal Development of the Hand and Wrist—A Radiographic Atlas and Digital Bone Age Companion,” published by Oxford University Press in 2011.

### 2.3 Model implementation

**FIGURE 1:**
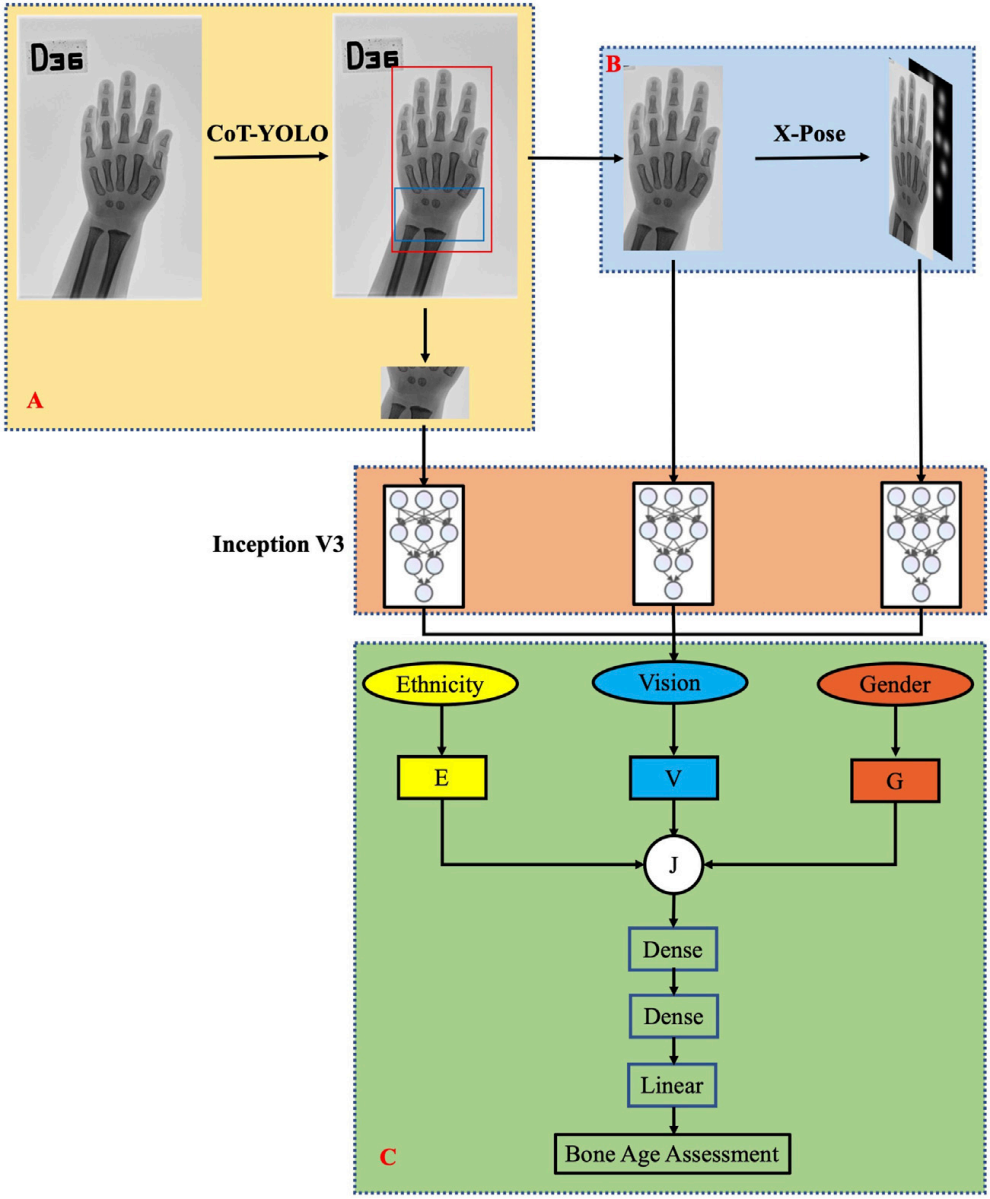
Schematic diagram of the EVG-BANet model. EVG-BANet comprises three modules: **(A)** CoT-YOLO for hand and wrist detection, **(B)** X-Pose for hand keypoint detection, and **(C)** BANet for BA prediction.

Our method is inspired by the clinical practice of radiologists, who consider various factors when assessing BA, including both global and local visual features extracted from hand radiographs, CA, gender, and ethnicity. In this work, we introduce EVG-BANet, a novel deep learning model that integrates all these factors to accurately and unbiasedly assess BA. The global visual features are extracted from hand radiographs, while the local visual features are extracted from anatomical regions of interest (ROIs) and wrist parts. The schematic diagram of EVG-BANet is shown in Figure 1.

EVG-BANet is a deep learning model that requires radiographs of the hand and wrist and information regarding the CA, gender, and ethnicity of children as inputs. It comprises three modules: CoT-YOLO for hand and wrist detection, X-Pose for hand keypoint detection, and BANet for BA prediction. The model was implemented using PyTorch 1.7.0, and the training was completed on NVIDIA GeForce RTX 2080 Ti GPUs. Here, we provide a detailed overview of the individual components of EVG-BANet. The table illustrating the dataset utilization by each module is shown in Supplementary Table S2.

CoT-YOLO: CoT-YOLO was constructed by integrating the YOLOv5 and Contextual Transformer (CoT) networks (Li et al., 2023). The schematic diagram of CoT-YOLO is shown in Supplementary Figure S1. Darknet-53 was used as the backbone network (Redmon and Farhadi, 2018). CoT can leverage contextual information to enhance visual representation (Li et al., 2023). Therefore, we used CoT before the convolutional layers and the BottleneckCSP module. During training, we used the pretrained model from the COCO dataset (Lin et al., 2014) and iteratively trained it for 5,000 epochs with an initial learning rate of 0.001. CoT-YOLO was trained on the training set from the local dataset and validated using the local test set.

X-Pose: To meet the challenge of radiograph-based hand pose estimation, we leveraged the recent state-of-the-art encoder–decoder architecture proposed by Eng et al. (2021) for human pose estimation. The schematic diagram of X-Pose is shown in Supplementary Figure S2. Specifically, X-Pose uses keypoints including the center of the wrist joint, finger tips, and interphalangeal joints. This model consists of a ResNet-50 backbone network followed by a series of deconvolutional layers. We trained the model initialized on ImageNet using the suggested training parameters for 20 epochs and the Adam optimizer, with a learning rate of 0.001. X-Pose was trained solely on the training set from the RHPE dataset, validated on the validation set from the RHPE dataset, and then, applied to the other datasets.

BANet: BANet uses the Inception v3 model (Szegedy et al., 2015) to extract features from three independent pathways: the hand bones identified using the CoT-YOLO model, the wrist bones identified using the CoT-YOLO model, and the Gaussian distribution attention map of hand keypoints. The three types of features extracted from the three pathways are combined using a mixed inception module, which is a neural network architecture that combines features from different layers of the network. Instead of simply concatenating the features, we introduce multipliers w_g_ and w_e_ to balance the relative importance of the inputs that are relevant to the final prediction. This allows our model to learn weighted representations of gender information G and ethnicity information E, according to their relevance to BAA. Information G and E can be represented as follows:

$$G=w_{g}\times g,$$

$$E=w_{e}\times e,$$

where g and e are the gender and ethnicity, respectively. Male gender is represented by g = 1, and female gender is represented by g = 0. Similarly, e = 1 denotes “Han” ethnicity, and e = 0 represents “Tibetan.” E and G are then concatenated with the visual feature V to form the feature J [J = (V; E; G)]. Finally, feature J is fed into two dense layers with ReLU activation functions to regress to the BA using an L1 loss. The Adam optimizer is used for training. The model is trained for 150 epochs using a batch size of 16. The initial learning rate is 0.001, and it is reduced by 20% every four epochs. The training of BANet relies on the training sets from the RSNA dataset, the RHPE dataset, and the local dataset. The validation of BANet is conducted using the validation sets from both the RSNA and RHPE datasets, as well as the test set from the local dataset.

EVG-BANet is the first BAA model to integrate visual features, gender, and ethnicity. This model has the potential to improve accuracy and reduce bias of BAA, particularly for children from diverse ethnicities.

### 2.4 Statistical analysis

Descriptive statistics were used to summarize the data as appropriate. Continuous variables were expressed as mean ± standard deviation (SD). Categorical variables were expressed as frequency and proportion. The children were categorized into three CA groups: group 1 (0–6 years old), group 2 (7–12 years old), and group 3 (13–18 years old).

To evaluate the accuracy and generalizability of EVG-BANet, an open-source state-of-the art model BoNet (Escobar et al., 2019) was used as a reference for comparison, and the overall performance of the two models was assessed by comparing the mean absolute difference (MAD) between the model estimates and the ground truth BA on the four datasets. BA accuracy, defined as the percentage of MAD within 1 year, was also compared. To determine agreement between models’ estimates and the ground truth BA, Bland–Altman plots were generated to show the difference between the estimates and the ground truth BA over the range of the mean of the two BA values. A paired *t*-test was used to compare the MAD between the two models. McNemar’s test was used to compare the accuracy of the two models. An ablation analysis was conducted to assess the impact of each module on the model performance. To evaluate the bias of EVG-BANet, the MADs between various demographic groups were compared using the *t*-test or analysis of variance. Scatterplots with the ground truth BA and model estimates on the horizontal and vertical axes, respectively, with superimposed identity lines (slope = 1 and intercept = 0) were generated to visualize differences between EVG-BANet predictions and the ground truth.

All analyses were performed using R version 4.2.2 (R Core Team, 2014). Statistical significance was defined as a two-tailed *p*-value of <0.05.

## 3 Results

### 3.1 Demographic characteristics

**FIGURE 2:**
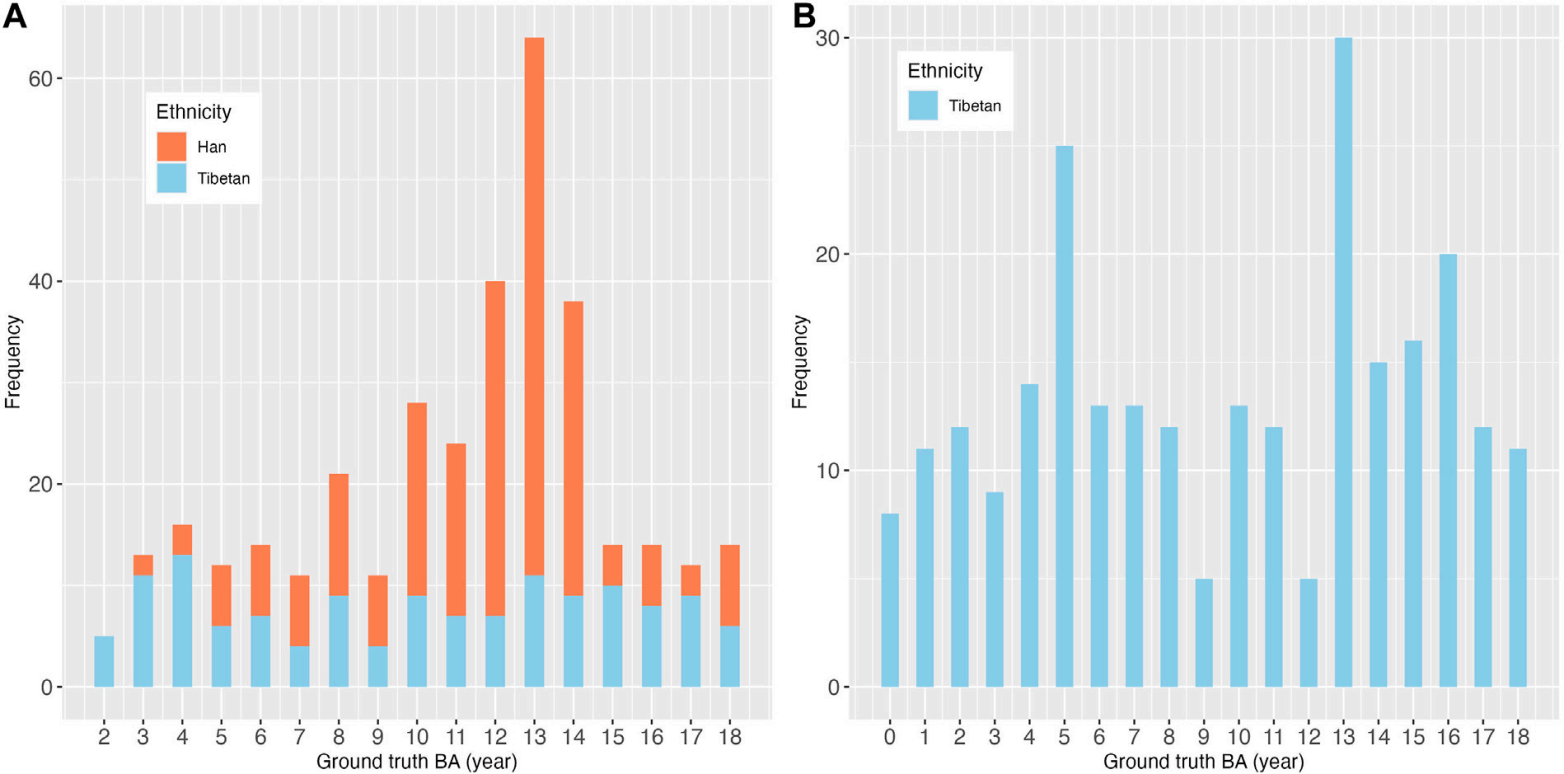
Distribution of ground truth BA and ethnicity composition of **(A)** the local test set and **(B)** the external test set.

The summary information for the four datasets is shown in Table 1. A total of 21951 radiographs were evaluated in this study, including 18927 training, 2138 validation, and 886 test images. Table 2 shows the demographic characteristics of the local and external test sets. In the local test set, the CA and the ground truth BA of the children were 11.71 ± 3.98 and 11.30 ± 3.97 years, respectively. The male-to-female ratio was 1.34:1 (57%:43%). The test set included two ethnic groups: Han and Tibetan, with a ratio of 1.6:1 (67%:33%). In the external test set, the CA and the ground truth BA were 11.30 ± 5.30 and 9.79 ± 5.29 years, respectively. The male-to-female ratio was 1.49:1 (60%:40%). All the children were Tibetan. Figure 2 shows the distribution of the ground truth BA and ethnicity composition for both the local and external test sets.

**TABLE 2:** Demographic characteristics of the local and external test sets.

Characteristic	Local test set			External test set
	Total (*n* = 351)	Han (*n* = 216)	Tibetan (*n* = 135)	Tibetan (*n* = 256)
Gender				
Male	201 (57.3%)	110 (50.9%)	91 (67.4%)	153 (59.8%)
Female	150 (42.7%)	106 (49.1%)	44 (32.6%)	103 (40.2%)
Chronological age group^a^				
Group 1	44 (12.5%)	12 (5.6%)	32 (23.7%)	64 (25.0%)
Group 2	161 (45.9%)	110 (50.9%)	51 (37.8%)	75 (29.3%)
Group 3	146 (41.6%)	94 (43.5%)	52 (38.5%)	117 (45.7%)
Chronological age (years)	11.71 ± 3.98	12.22 ± 3.54	10.90 ± 4.49	11.30 ± 5.30
Bone age (years)	11.30 ± 3.97	11.88 ± 3.09	10.37 ± 4.95	9.79 ± 5.29

^a^ The children were categorized into three chronological age groups: group 1 (0–6 years old), group 2 (7–12 years old), and group 3 (13–18 years old).

### 3.2 Comparison of accuracy and generalizability between EVG-BANet and BoNet

**RSNA and RHPE test sets:** In the RSNA test set, EVG-BANet and BoNet exhibited comparable performance with an MAD of 0.34 ± 0.16 and 0.35 ± 0.18 years, respectively (*p* = 0.934). In the RHPE test set, EVG-BANet achieved an MAD of 0.52 ± 0.21 years, which was significantly more accurate than that of BoNet (0.63 ± 0.23 years, *p* < 0.001).

**FIGURE 3:**
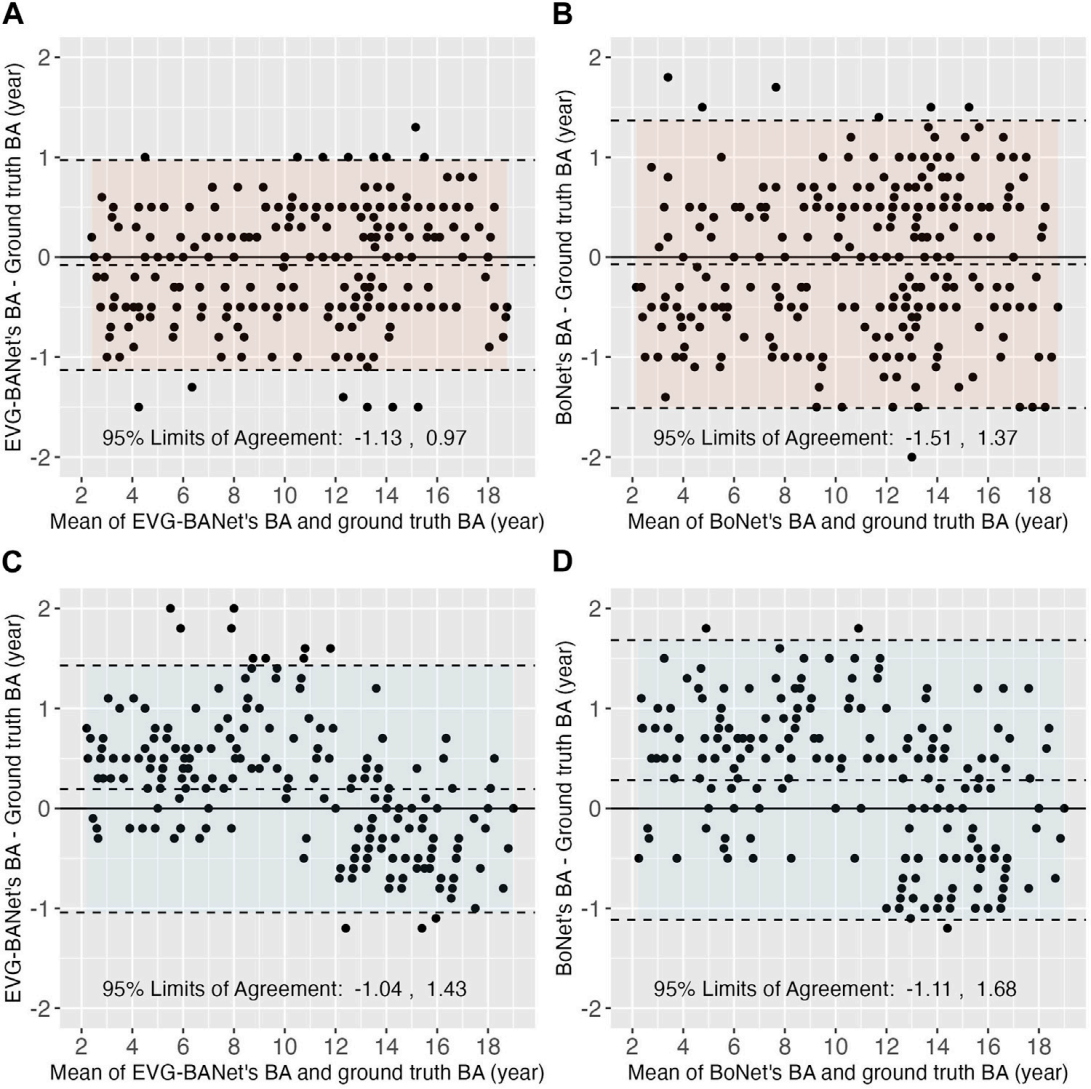
Bland–Altman plots showing the differences between the estimated BA by EVG-BANet/BoNet and the ground truth BA. In the local test set, the Bland–Atman plots for **(A)** EVG-BANet and **(B)** BoNet are shown; in the external test set, the Bland–Atman plots for **(C)** EVG-BANet and **(D)** BoNet are illustrated.

**Local test set:** In the local test set, EVG-BANet exhibited superior performance compared to BoNet, both in the overall assessment and within the subgroups stratified by CA, gender, and ethnicity (Table 3). Overall, EVG-BANet achieved an MAD of 0.47 ± 0.28 years, significantly outperforming BoNet with an MAD of 0.62 ± 0.40 years (*p* < 0.001); moreover, the accuracy within 1 year of EVG-BANet was 97.7%, while that of BoNet was 90% (*p* < 0.001) (Supplementary Table S3). The 95% limits of agreement for EVG-BANet and the ground truth BA were −1.13 to 0.97 years, according to the Bland–Altman plot (Figure 3A), while the limits of agreement for BoNet and the ground truth BA were −1.51 to 1.37 years (Figure 3B).

**TABLE 3:** Comparison of the MAD of EVG-BANet and BoNet for the local and external test sets.

Characteristic	MAD (years) of the local test set			MAD (years) of the external test set		
	EVG-BANet	BoNet	*p*-value	EVG-BANet	BoNet	*p*-value
**Total**	0.47 ± 0.28	0.62 ± 0.40	<0.001	0.53 ± 0.39	0.66 ± 0.40	<0.001
Gender						
Male	0.47 ± 0.29	0.61 ± 0.38	<0.001	0.57 ± 0.43	0.68 ± 0.41	<0.001
Female	0.46 ± 0.26	0.64 ± 0.41	<0.001	0.48 ± 0.31	0.63 ± 0.37	<0.001
Chronological age group^a^						
Group 1	0.48 ± 0.27	0.68 ± 0.40	<0.001	0.42 ± 0.29	0.60 ± 0.38	<0.001
Group 2	0.45 ± 0.28	0.59 ± 0.41	<0.001	0.69 ± 0.48	0.76 ± 0.47	0.011
Group 3	0.48 ± 0.28	0.64 ± 0.39	<0.001	0.49 ± 0.34	0.62 ± 0.34	<0.001
Ethnicity						
Han	0.47 ± 0.26	0.60 ± 0.36	<0.001	/	/	/
Tibetan	0.46 ± 0.30	0.66 ± 0.44	<0.001	0.53 ± 0.39	0.66 ± 0.40	<0.001

^a^ The children were categorized into three chronological age groups: group 1 (0–6 years old), group 2 (7–12 years old), and group 3 (13–18 years old).

To assess the individual impact of each module on the performance of EVG-BANet, an ablation analysis was conducted on the local test set (Supplementary Table S4). The omission of visual features from wrist and keypoints resulted in a decrease in accuracy within 1 year by 6% and 8.2%, respectively. The exclusion of ethnicity information led to a reduction in accuracy by 3.2%.

**External test set:** In the external test set, EVG-BANet demonstrated a significantly lower MAD than BoNet, both in total (0.53 ± 0.39 vs. 0.66 ± 0.40 years, *p* < 0.001) and within the demographic subgroups (Table 3). The accuracy within 1 year of EVG-BANet was higher than that of BoNet in total (89.5% vs. 85.5%, *p* = 0.066) and within the subgroups, although the difference was not statistically significant (Supplementary Table S3). According to the Bland–Altman plot, the 95% limits of agreement for EVG-BANet and the ground truth BA were −1.04–1.43 years (Figure 3C), while those for BoNet and the ground truth BA were −1.11–1.68 years (Figure 3D).

### 3.3 Bias of EVG-BANet

**FIGURE 4:**
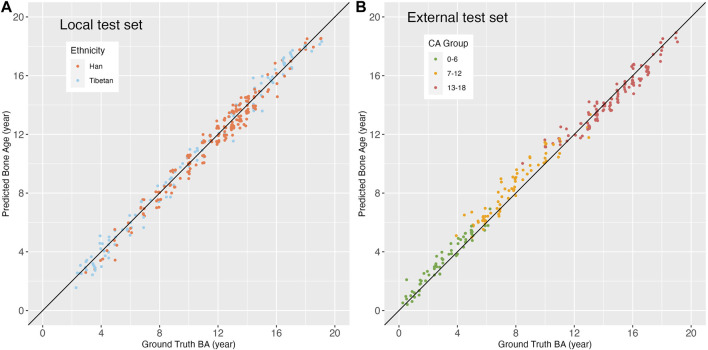
Scatterplots showing the differences between the ground truth BA and EVG-BANet prediction based on ethnicity and chronological age. **(A)** Scatterplot based on ethnicity in the local test set. **(B)** Scatterplot based on CA groups in the external test set. The identity line (black) has a slope and intercept of 1 and 0, respectively.

**Local test set:** EVG-BANet showed no significant bias in the local test set. Regarding gender, CA group, and ethnicity, no significant differences were observed in the MAD of EVG-BANet across the subgroups [male, 0.47 ± 0.29 years vs. female, 0.46 ± 0.26 years (*p* = 0.794); group 1, 0.48 ± 0.27 years vs. group 2, 0.45 ± 0.28 years vs. group 3, 0.48 ± 0.28 years (*p* = 0.764); Han, 0.47 ± 0.26 years vs. Tibetan, 0.46 ± 0.30 years (*p* = 0.848)]. The MAD of EVG-BANet between the Han and Tibetan populations was further compared across the different subgroups, and no bias was detected (Table 4). The scatterplot illustrating the differences between the ground truth BA and EVG-BANet predictions in the Han and Tibetan populations did not show a clear tendency of bias (Figure 4A). No bias based on CA (Supplementary Figure S3) or gender (Supplementary Figure S4A) was found.

**TABLE 4:** The MAD of EVG-BANet stratified by demographic groups and ethnicity in the local test set.

Characteristic	MAD (years) of the local test set		
	Han	Tibetan	*p*-value
**Total**	0.47 ± 0.26	0.46 ± 0.30	0.848
Gender			
Male	0.48 ± 0.29	0.46 ± 0.28	0.695
Female	0.46 ± 0.23	0.47 ± 0.34	0.892
Chronological age group^a^			
Group 1	0.45 ± 0.22	0.48 ± 0.28	0.706
Group 2	0.47 ± 0.28	0.41 ± 0.27	0.186
Group 3	0.46 ± 0.26	0.50 ± 0.33	0.484

^a^ The children were categorized into three chronological age groups: group 1 (0–6 years old), group 2 (7–12 years old), and group 3 (13–18 years old).

**External test set:** In the external test set, the MAD of EVG-BANet showed no significant difference between male and female subgroups (0.57 ± 0.43 vs. 0.48 ± 0.31 years, *p* = 0.068); however, a significant difference was noted among the different CA groups [group 1, 0.42 ± 0.29 years vs. group 2, 0.69 ± 0.48 years vs. group 3, 0.49 ± 0.34 years (*p* < 0.001)]. The scatterplot indicated a tendency where the BA assessed by EVG-BANet was overestimated in children aged 0–6 years and 7–12 years, while it was underestimated in children aged 13–18 years (Figure 4B). No bias based on gender was observed (Supplementary Figure S4B).

## 4 Discussion

We developed a fully automated deep learning model named EVG-BANet for BAA based on the GP method. EVG-BANet was trained using radiographs from three distinct populations, one of which included both Han and Tibetan ethnic groups. This model incorporates ethnicity and gender as independent variables and integrates them with the global and local features extracted from radiographs for the final BA prediction. In all four test sets, including an external test set, EVG-BANet exhibited superior performance compared to BoNet.

Overall, EVG-BANet demonstrated good accuracy and generalizability. EVG-BANet was trained using 18927 images (12611 from the RSNA dataset, 5491 from the RHPE dataset, and 825 from the local dataset) and validated using 2138 images (1425 from the RSNA dataset and 713 from the RHPE dataset). EVG-BANet showed MAD values of 0.34, 0.52, and 0.47 years for the RSNA test set, RHPE test set, and local test set (including 216 Han children and 135 Tibetan children), respectively, and 0.53 years for the external test set (including 256 Tibetan children). The model achieved an accuracy within 1 year of 97.7% and 89.5% in the local and external test sets, respectively. In both the overall evaluation and evaluation within the various demographic groups (including gender, CA, and ethnicity), EVG-BANet consistently outperformed BoNet, with lower MAD and higher accuracy within 1 year.

We conducted ablation studies to assess the individual contributions of key modules within EVG-BANet on the local test set. Visual features from both wrist and keypoints significantly contribute to accuracy. The ethnicity information serves a complementary role in tailoring predictions to diverse populations. Recently, Nguyen et al. (2022) developed a BAA approach that integrates keypoint detection and gender information; however, our model integrates a more comprehensive array of visual and demographic features. To the best of our knowledge, EVG-BANet is the first BAA approach that combines both global and local visual features with crucial demographic factors such as gender and ethnicity. Global features are derived from the entire hand radiograph using a convolutional neural network, while local features are extracted from specific anatomical ROIs and wrist segments using a hand keypoint detection model. The coexistence of both global and local features in an assessment model is essential for accurate BAA, as global features provide insights into the overall development of the hand and wrist, while local features capture the fine-grained details of individual bones. EVG-BANet also considers ethnic information along with visual features. Previous studies have shown that conventional BAA methods can lead to substantial disparities in outcomes across different ethnic groups (Ontell et al., 1996; Zhang et al., 2009). EVG-BANet addresses this inherent bias by incorporating ethnicity into its model, which enables it to better account for these differences and improve the accuracy of BAA. The model exhibited good generalization in the external test set, which has markedly different demographic characteristics (e.g., CA distribution, gender ratio, altitude, and ethnic composition) compared to the training dataset.

Prior to this study, none of the models included the Tibetan population as part of the training set. The initial AI BAA models were primarily developed using North American and Korean populations (Kim et al., 2017; Lee et al., 2017; Larson et al., 2018; Mutasa et al., 2018), followed by studies based on the Chinese population (Ren et al., 2019; Zhou et al., 2020; Yang et al., 2023). However, the Chinese population-based models predominantly focused on the Han ethnic group and did not include the Tibetan ethnic group. Tibetan people mainly reside in the Tibetan Plateau of western China, where the average altitude is as high as 4000 m; consequently, this region has limited healthcare facilities because of its unique geographical location (Harris et al., 2001; Wang et al., 2021). Our model could assess BA accurately in Tibetan children, thus demonstrating its great potential in assisting radiologists, pediatricians, and endocrinologists to conduct accurate BAA with less labor cost and shorter time in Tibet.

Biases of EVG-BANet against specific demographic groups were also evaluated. EVG-BANet showed no bias based on gender, CA, or ethnicity in the local test set. However, in the external test set, EVG-BANet tended to overestimate BA in children aged 0–12 years and underestimate BA in children aged 13–18 years. BoNet also exhibited a similar tendency (data not shown). This is possibly due to the following two reasons: first, the appearance and development of ossification centers show considerable variation in younger children, while the wrist bones are matured in adolescents; the determination of BA mainly relies on the fusion stage of the epiphysis of the phalanges and metacarpals. AI models might focus on different maps as compared to human readers (Spampinato et al., 2017). Second, the external test set included a population that resides at an ultrahigh altitude of 4500 m asl; the development of BA in children residing at this altitude might be different from that in children residing in an area at 3600 m asl altitude and in plain areas (Cidanwangjiu et al., 2023). Therefore, this bias should be carefully monitored and corrected in the future application of the AI BA systems.

Our study has some limitations. First, similar to previous studies on BAA, there is no gold standard for BA evaluation. The ground truth BA used for training is determined by reviewers, which is inevitably influenced by inter- and intra-reviewer variations. Second, the ethnic composition of the external test set was exclusively Tibetan; therefore, we were unable to externally evaluate ethnic bias between the Han and Tibetan populations. Third, our model may not be able to identify certain disorders that a human reviewer could potentially detect from radiographic images, such as hypochondroplasia, rickets, and congenital syndromes (van Rijn and Thodberg, 2013). Fourth, our model could not handle missing values. We plan to investigate suitable strategies for the future versions of EVG-BANet.

## 5 Conclusion

In conclusion, we developed an AI BAA model termed EVG-BANet that estimates BA with high accuracy in both Han and Tibetan children. The EVG-BANet model could potentially enhance the efficiency and accuracy of BAA and can be applied in areas with limited medical resources, such as rural regions and the Tibetan plateau.

## Data Availability

The raw data supporting the conclusion of this article will be made available by the authors, without undue reservation.

## Supplementary material

Supplementary figure S1 Schematic diagram of CoT-YOLO.

Supplementary figure S2 Schematic diagram of X-Pose.

Supplementary figure S3 Scatterplot showing the differences between the ground truth BA and EVG-BANet prediction based on the chronological age groups for the local test set.

Supplementary figure S4 Scatterplots showing the differences between the ground truth BA and EVG-BANet prediction based on gender for **(A)** the local test set and **(B)** the external test set.
