# Supplementary figures and images for: An artificial intelligence-based bone age assessment model for Han and Tibetan children
Source: Front Physiol. 2024 Feb 15;15:1329145. doi: 10.3389/fphys.2024.1329145 (PMC10902452; doi:10.3389/fphys.2024.1329145)

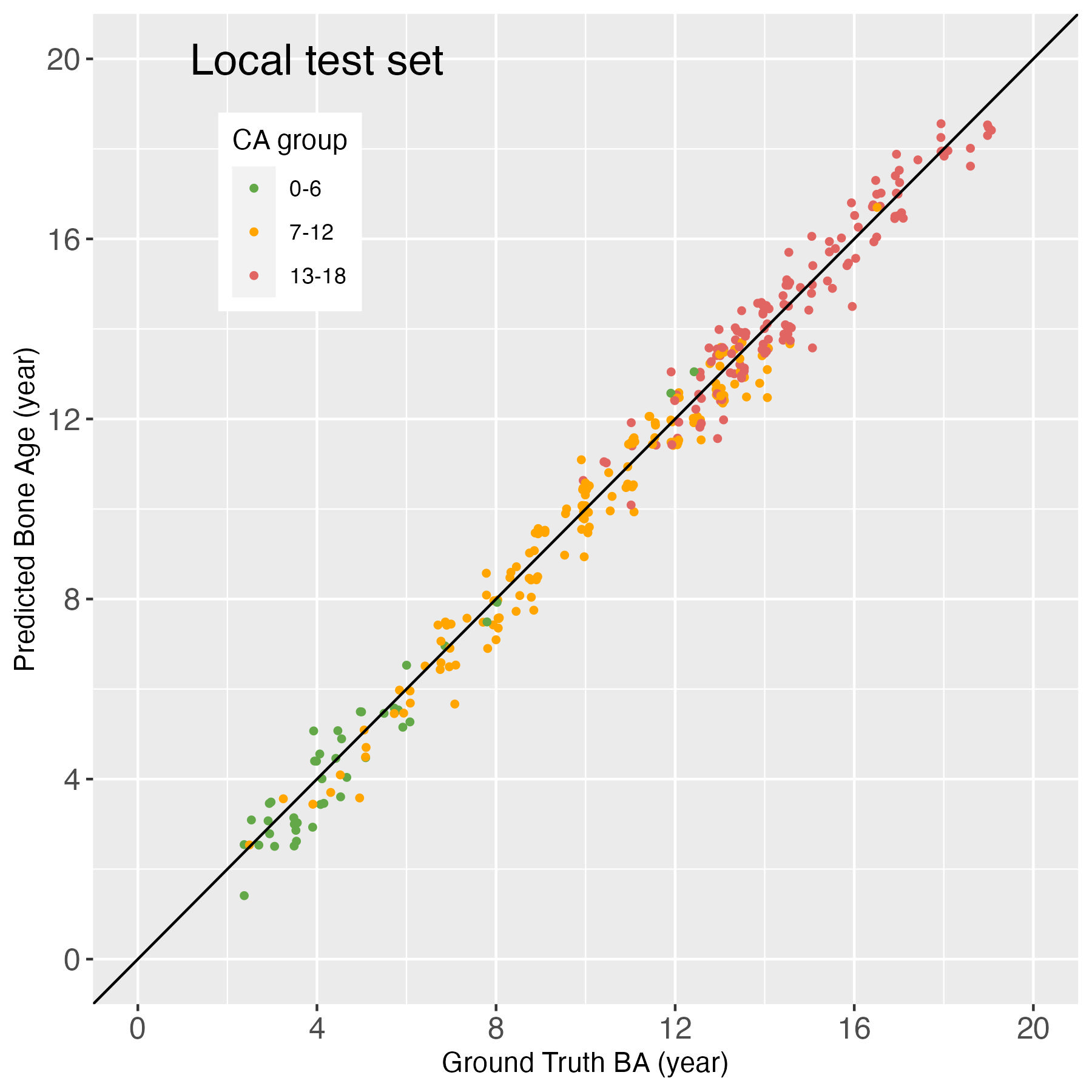

Supplement: Supplementary file 1 [file Image3.JPEG]

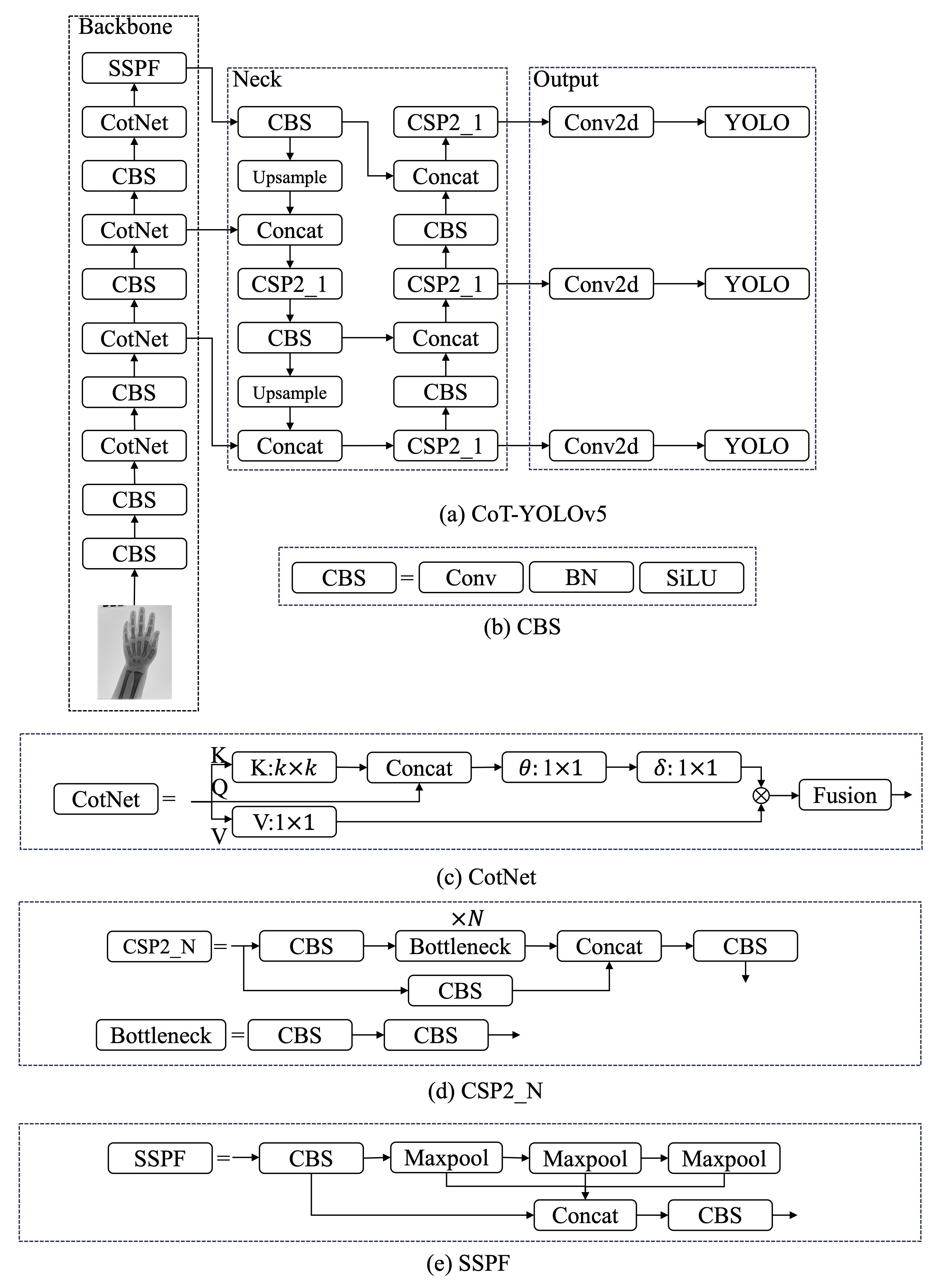

Supplement: Supplementary file 3 [file Image1.JPEG]

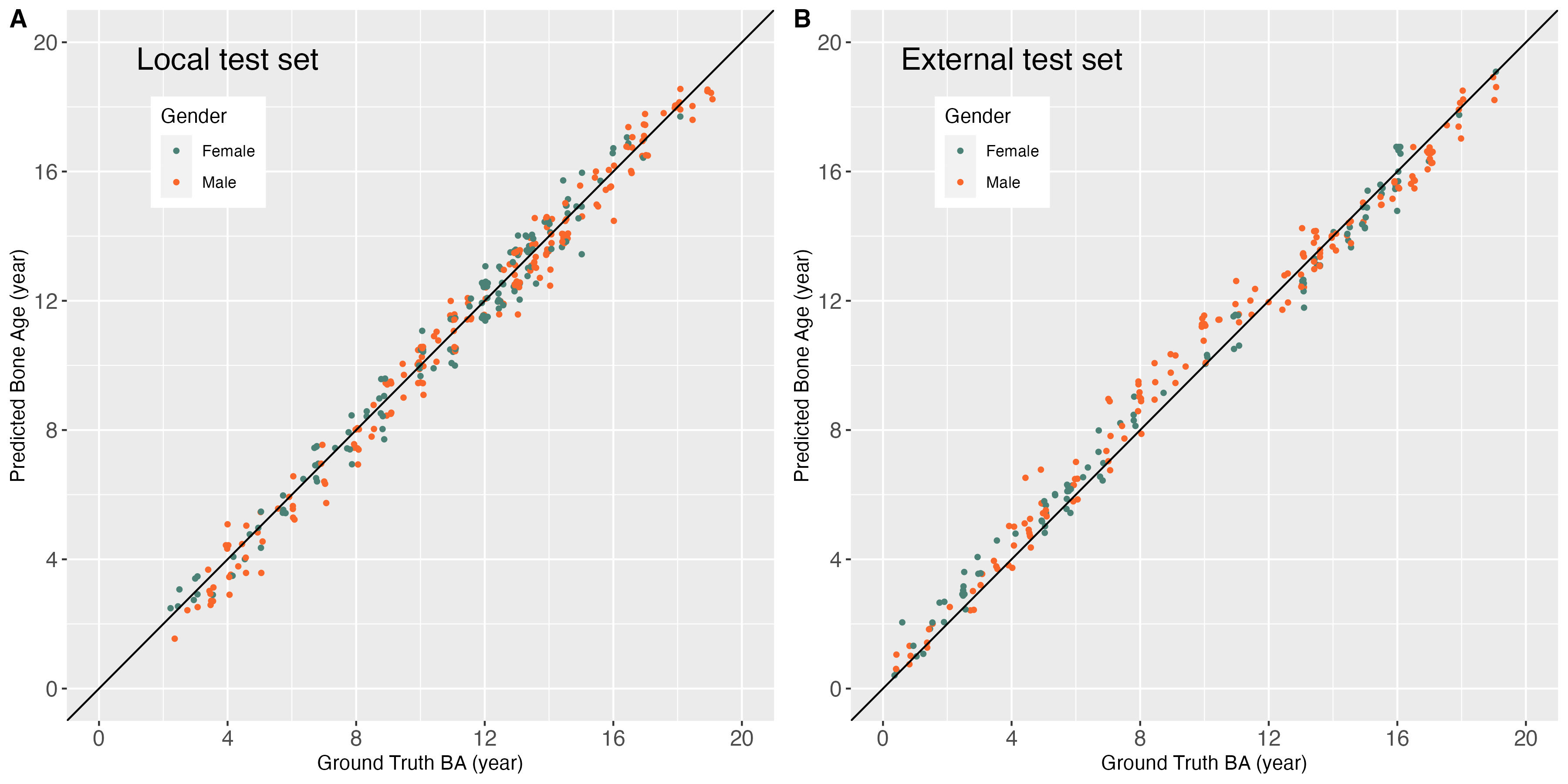

Supplement: Supplementary file 4 [file Image4.JPEG]

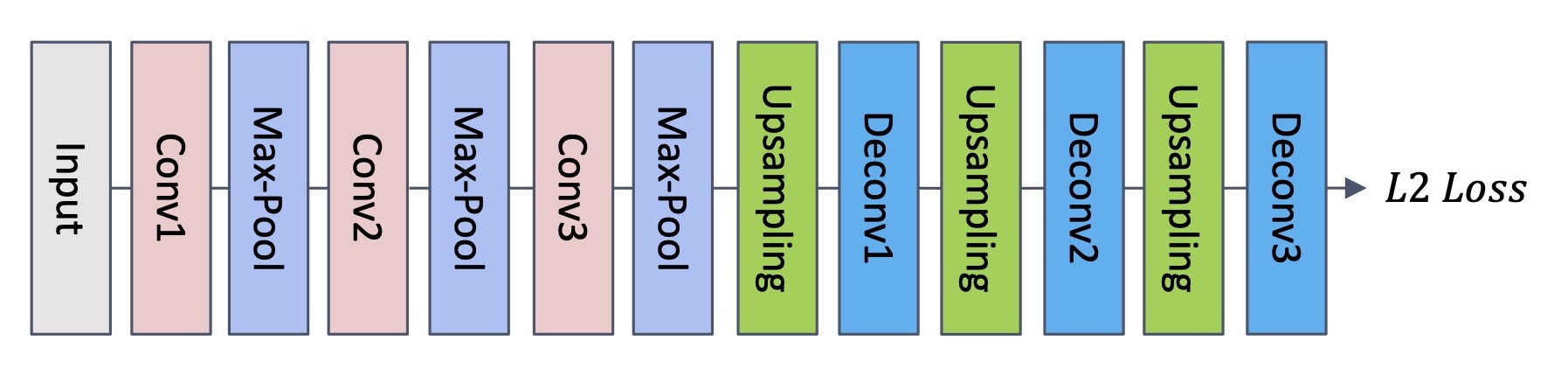

Supplement: Supplementary file 5 [file Image2.JPEG]
