# Supplementary material for: An artificial intelligence-based bone age assessment model for Han and Tibetan children
Source: Front Physiol. 2024 Feb 15;15:1329145. doi: 10.3389/fphys.2024.1329145 (PMC10902452; doi:10.3389/fphys.2024.1329145)
Supplement: Supplementary file 2 [file Table1.DOCX]

Supplementary Material

**Table S1** Inclusion and exclusion criteria for children in the external test set.

| Inclusion criteria | Exclusion criteria |
| --- | --- |
| (1) aged 0–18 years; | (1) age greater than 19 years; |
| (2) born and raised in the corresponding city; | (2) missing age, sex, or ethnicity information; |
| (3) presenting to the hospital for traumatic injury; | (3) presence of underlying systemic diseases that may impact BA development; |
| (4) having left hand-wrist radiographs taken in Nyima County People’s Hospital in Nagqu from September 2018 to July 2022. | (4) unable to meet the requirements of BA interpretation due to poor quality of X-ray images. |
