# Supplementary material for: An artificial intelligence-based bone age assessment model for Han and Tibetan children
Source: Front Physiol. 2024 Feb 15;15:1329145. doi: 10.3389/fphys.2024.1329145 (PMC10902452; doi:10.3389/fphys.2024.1329145)
Supplement: Supplementary file 6 [file Table2.DOCX]

Supplementary Material

**Table S2** Dataset utilization by each module in EVG-BANet.

| Module | Dataset | Training (n) | Validation (n) |
| --- | --- | --- | --- |
| CoT-YOLO | Local | 825 | 351 |
| X-Pose | RHPE | 5491 | 713 |
| BANet | RSNA | 12611 | 1425 |
|  | RHPE | 5491 | 713 |
|  | Local | 825 | 351 |

CoT-YOLO is trained and validated solely on the local dataset. X-Pose is trained and validated solely on the RHPE dataset. BANet is trained and validated on all three datasets. Due to the limited size of the local dataset, it is categorized into a training set and a test set. The local test set is utilized to validate CoT-YOLO and BANet.
