# Supplementary material for: An artificial intelligence-based bone age assessment model for Han and Tibetan children
Source: Front Physiol. 2024 Feb 15;15:1329145. doi: 10.3389/fphys.2024.1329145 (PMC10902452; doi:10.3389/fphys.2024.1329145)
Supplement: Supplementary file 7 [file Table3.DOCX]

Supplementary Material

**Table S3** Comparison of accuracy within 1 year of EVG-BANet and BoNet for the local and external test sets.

| Characteristic | Accuracy within 1 year n (%)  of the local test set | | | Accuracy within 1 year n (%)  of the external test set | | |
| --- | --- | --- | --- | --- | --- | --- |
|  | EVG-BANet | BoNet | P-value | EVG-BANet | BoNet | P-value |
| **Total** | 343/351 (97.7%) | 316/351 (90.0%) | <0.001 | 229/256 (89.5%) | 219/256 (85.5%) | 0.066 |
| **Gender** |  |  |  |  |  |  |
| Male | 195/201 (97.0%) | 182/201 (90.5%) | 0.002 | 133/153 (86.9%) | 131/153 (85.6%) | 0.752 |
| Female | 148/150 (98.7%) | 134/150 (89.3%) | <0.001 | 96/103 (93.2%) | 88/103 (85.4%) | 0.061 |
| **Chronologic age group*** |  |  |  |  |  |  |
| Group1 | 44/44 (100%) | 39/44 (88.6%) | 0.074 | 61/64 (95.3%) | 56/64 (87.5%) | 0.074 |
| Group2 | 157/161 (97.5%) | 147/161 (91.3%) | 0.004 | 59/75 (78.7%) | 57/75 (76.0%) | 0.724 |
| Group3 | 142/146 (97.3%) | 130/146 (89.0%) | 0.003 | 109/117 (93.2%) | 106/117 (90.6%) | 0.547 |
| **Ethnicity** |  |  |  |  |  |  |
| Han | 212/216 (98.1%) | 196/216 (90.7%) | <0.001 | / | / | / |
| Tibetan | 131/135 (97.0%) | 120/135 (88.9%) | 0.006 | 229/256 (89.5%) | 219/256 (85.5%) | 0.066 |

* The children were categorized into three chronological age groups: Group 1 (0–6 years old), Group 2 (7–12 years old), and Group 3 (13–18 years old).
