# Supplementary material for: An artificial intelligence-based bone age assessment model for Han and Tibetan children
Source: Front Physiol. 2024 Feb 15;15:1329145. doi: 10.3389/fphys.2024.1329145 (PMC10902452; doi:10.3389/fphys.2024.1329145)
Supplement: Supplementary file 8 [file Table4.DOCX]

Supplementary Material

**Table S4** Ablation studies to assess the individual contributions of key modules within EVG-BANet on the local test set

| Feature Removed | Accuracy within 1 Year (%) | Change from EVG-BANet |
| --- | --- | --- |
| None (Baseline) | 343/351 (97.7%) | N/A |
| Wrist | 322/351 (91.7%) | -6.0% |
| Keypoints | 314/351 (89.5%) | -8.2% |
| Ethnicity | 332/351 (94.6%) | -3.1% |
